# Comment on Megat Ramli et al. A Systematic Review: The Role of Artificial Intelligence in Lung Cancer Screening in Detecting Lung Nodules on Chest X-Rays. *Diagnostics* 2025, *15*, 246

**DOI:** 10.3390/diagnostics16010109

**Published:** 2025-12-29

**Authors:** Aybaniz Ismayilli, Jan Hansel, Emmanuel Erhieyovwe

**Affiliations:** 1 Research and Innovation, Manchester University NHS Foundation Trust, Manchester M13 9WL, UK; emmanuel.erhieyovwe@mft.nhs.uk; 2 Division of Immunology, Immunity to Infection and Respiratory Medicine, The University of Manchester, Manchester M13 9WL, UK; jan.hansel@manchester.ac.uk

We read with interest the review by Megat Ramli et al. [1], which systematically compares artificial intelligence (AI) with radiologists for lung-nodule detection on chest x-rays (CXR). The review explores factors that influence performance (size, morphology, location) and summarizes patterns of missed nodules. The review also provides a summary of AI performance, noting that AI frequently achieves a higher performance than radiologists [1].

The review correctly notes that high sensitivity of AI can be accompanied by an increased number of false-positive findings. In settings where prevalence is low, even a small increase in the false-positive rate can translate into a significant burden on healthcare resources, leading to unnecessary follow-up computed tomography (CT) scans, additional clinic visits, and heightened patient anxiety [2]. While the review addresses the technical performance of AI, it does not fully explore how the high false-positive rate can offset the benefits of improved sensitivity.

The true value of AI in a clinical setting must be measured not just by its algorithmic performance but by its overall impact on the healthcare system and patient outcomes. We believe that future reviews and studies should move beyond a sole focus on sensitivity and specificity and provide a more thorough analysis of the downstream effects of AI-generated false positives. This would provide a complete and more accurate picture of AI’s true role in lung cancer screening. This analysis can be achieved by using specific study designs, such as stepped-wedge cluster randomized trials, which assess the real-world impact of AI on clinical workflow and downstream patient outcomes—as demonstrated in a recent study protocol [3].

To align technical performance with clinical value, we suggest readers designing or evaluating services may wish to assure the following principles. AI should be used in an assistive rather than autonomous manner, deployed as a second reader, with particular attention to commonly missed zones [4]. Operating thresholds should be balanced and clearly documented, ensuring that sensitivity gains are not achieved at the cost of disproportionate false positives [5]. Routine monitoring should be implemented with simple recording of the actions following AI alerts (such as further imaging or clinic review) to make workload and patient impact visible [6]. Reporting should be stratified by nodule size, morphology, and anatomical location, reflecting where performance differs [1].

The above are general reporting and governance habits. They help readers interpret sensitivity and specificity alongside practical consequences without introducing new data. AI can add real value as an assistive tool, especially for readers with less experience, but variability and activity arising from false-positive findings must be pro-actively managed. Presenting technical accuracy together with clear information on downstream actions and workload will offer a more complete picture of the role of AI in lung cancer screening.

## Abbreviations

The following abbreviations are used in this manuscript:

AI	artificial intelligence
CXR	chest X-rays
CT	computed tomography
